# Peripheral perfusion noninvasive monitoring technologies - a literature and patent review

**DOI:** 10.1007/s10877-026-01412-4

**Published:** 2026-02-02

**Authors:** Irene Orellana Plaza, Jenny Dankelman, Jan Bakker

**Affiliations:** 1 Department of Biomechanical Engineering, Delft University of Technology, Delft, The Netherlands; 2 Department of Intensive Care Adults, Erasmus MC University Medical Center, Rotterdam, The Netherlands

**Keywords:** Peripheral perfusion, Shock, Monitoring technologies, Hemodynamic parameters

## Abstract

Shock is a life-threatening condition marked by inadequate tissue perfusion and oxygen supply, leading to organ failure if not rapidly addressed. Clinical management of shock involves detecting and correcting altered macro hemodynamic parameters. However, these parameters may not accurately reflect microcirculatory alterations or abnormalities in oxygenation. A resuscitation strategy focused on peripheral perfusion, which can be non-invasively monitored, may allow for earlier shock detection and treatment, potentially reducing mortality. This literature review aims to study the available technologies found in literature and in patents to non-invasively monitor peripheral perfusion. PRISMA method was employed to systematically select or exclude articles and patents, resulting in 44 studies and 21 patents included in the review. The found technologies were classified based on the sensing principle in light (reflected, transmitted, or scattered), Doppler effect, temperature, and skin mottling. Combining the monitorization of microcirculatory with macrocirculatory parameters has the potential to have an accurate prognosis value for shock and other diseases. However, the various technologies that have been developed to monitor peripheral perfusion require further research and testing in diverse conditions.

## Introduction

Shock is a life-threatening condition defined by inadequate tissue perfusion and thus oxygen supply, resulting in organ failure when not corrected rapidly [1]. In 2017, sepsis affected 48.9 million people and caused 11 million deaths globally [2]. Specifically, septic shock, the most common type of shock, accounted for 19.7% of all global deaths.

Shock diagnosis is mainly focused on macro hemodynamic parameters, such as blood pressure, heart rate, and serum lactate levels [1, 3]. Treatment typically targets the macro hemodynamic parameters through fluid resuscitation and the use of vasoactive medications. However, global hemodynamic parameters poorly reflect alterations at the microcirculatory level, so abnormal microcirculatory perfusion, and thus oxygenation, may persist despite correction of these macrocirculatory parameters [4, 5].

In the early stages of shock, compensatory mechanisms are activated to preserve perfusion to vital organs. These mechanisms involve the redistribution of blood flow away from less critical tissues, such as the skin and extremities, toward central organs, resulting in clinical signs of poor peripheral perfusion [6]. Several studies have demonstrated a relationship between peripheral perfusion alterations and organ dysfunction [7, 8]. Consequently, a resuscitation strategy that targets peripheral perfusion, rather than macro-hemodynamic parameters, holds the potential to reduce organ dysfunction and mortality [9, 10].

Peripheral perfusion can be non-invasively monitored based on clinical assessment by analyzing skin mottling and temperature or by measuring capillary refill time (CRT) [11]. CRT is the time required for color to return to a distal capillary bed after pressure is applied to cause blanching [11]. These clinical assessments are, however, subjective and may vary between health professionals. Therefore, in recent years, efforts have been made to develop technologies that monitor peripheral perfusion more objectively. This literature review aims to collect and analyze the available technologies that can be employed to non-invasively monitor peripheral perfusion. Due to CRT’s simplicity and clinical relevance, a special attention has been given to devices measuring CRTs, and a patent review on it has been included as well.

## Method

### Literature search method

The Preferred Reporting Items for Systematic Reviews and Meta-Analyses (PRISMA) method was employed to systematically select and exclude articles [12]. The search was conducted in four different databases (Scopus, PubMed, IEEE, and University Carlos III of Madrid (UC3M) Library) applying a single query (*“capillary refill time” OR “peripheral perfusion”)* in each database. To obtain an overview of the current relevant technologies only the articles published in the last 10 years were included. One author (IOP) performed the initial screening of titles, abstract and full texts. A second author (JD) double checked whether exclusion of articles was justified by screening their titles, abstracts, and full texts.

### Eligibility criteria

The goal of this review is to create an overview of technologies that can be used to non-invasively monitor peripheral perfusion, therefore, articles that describe invasive techniques to monitor peripheral perfusion were excluded. Articles about disease diagnoses, treatments or clinical studies that mention peripheral perfusion without focusing on or explaining the technology itself were also not included. Furthermore, only devices that were designed to measure in humans were included; during the article title evaluation articles about dogs (60), horses (32), and other animals such as chimpanzees or penguins were found. Finally, due to the anatomical differences between adults and babies, papers solely focusing on monitoring peripheral perfusion in newborns were also excluded.

### Patent search

Again, the PRISMA method was used to find patented medical devices that describe technology to measure CRT by using the query “capillary refill time” with a full text search in the Espacenet patent database. Patents not describing CRT measuring technologies (e.g. CRT as a part of a disease diagnosis) were excluded. Furthermore, medical devices that measure CRT but are not specifically designed to measure CRT (e.g. a device to measure two or more cardio-respiratory parameters and one of the embodiment measures CRT) were not included. This was done to narrow the search and have more specific results about CRT technology. Furthermore, patents that describe similar devices and have the same author were considered duplicated, and only the most recent version was included.

## Results

**Fig. 1:**
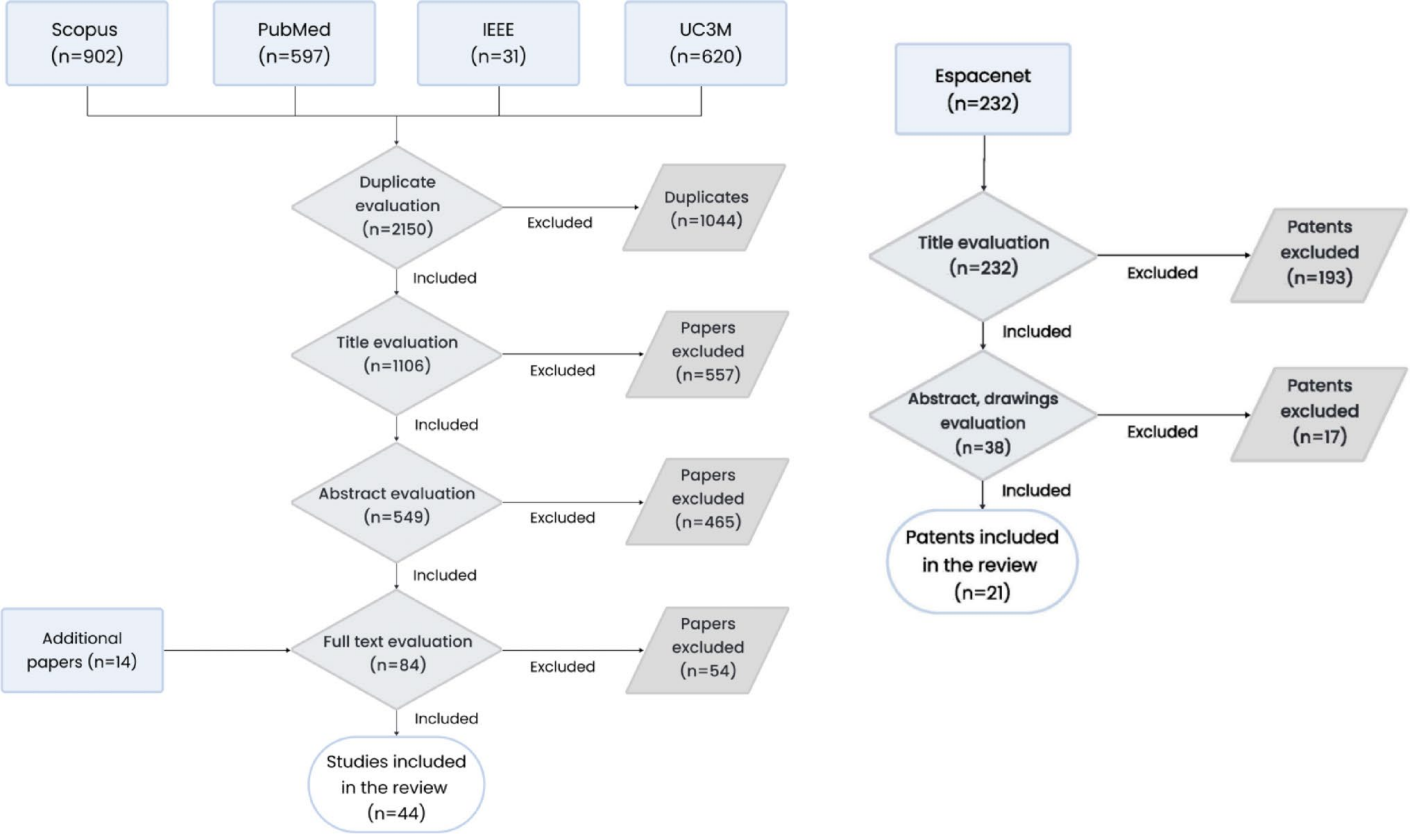
Schematic representation of the articles selection method. Left for articles, right for patents

The initial literature search resulted in the identification of 1106 non-duplicated articles. PubMed, IEEE, and UC3M databases’ articles were compared with Scopus’ articles, obtaining 900 non-duplicated articles from Scopus, 140 from PubMed, 14 from IEEE and 216 from the UC3M database. After title, abstract, and content screening, 40 papers were included. Additional 14 papers found through other papers’ references were added. Therefore, a total of 44 articles were included in the review (Fig. 1, **left**). The initial research in Espacenet resulted in 232 patens. After screening a total of 21 were included in the review **(**Fig. 1, **right).**

*The databases used for the search are shown at the top and the total number of studies after the PRISMA method application at the bottom of the scheme*.

**Fig. 2:**
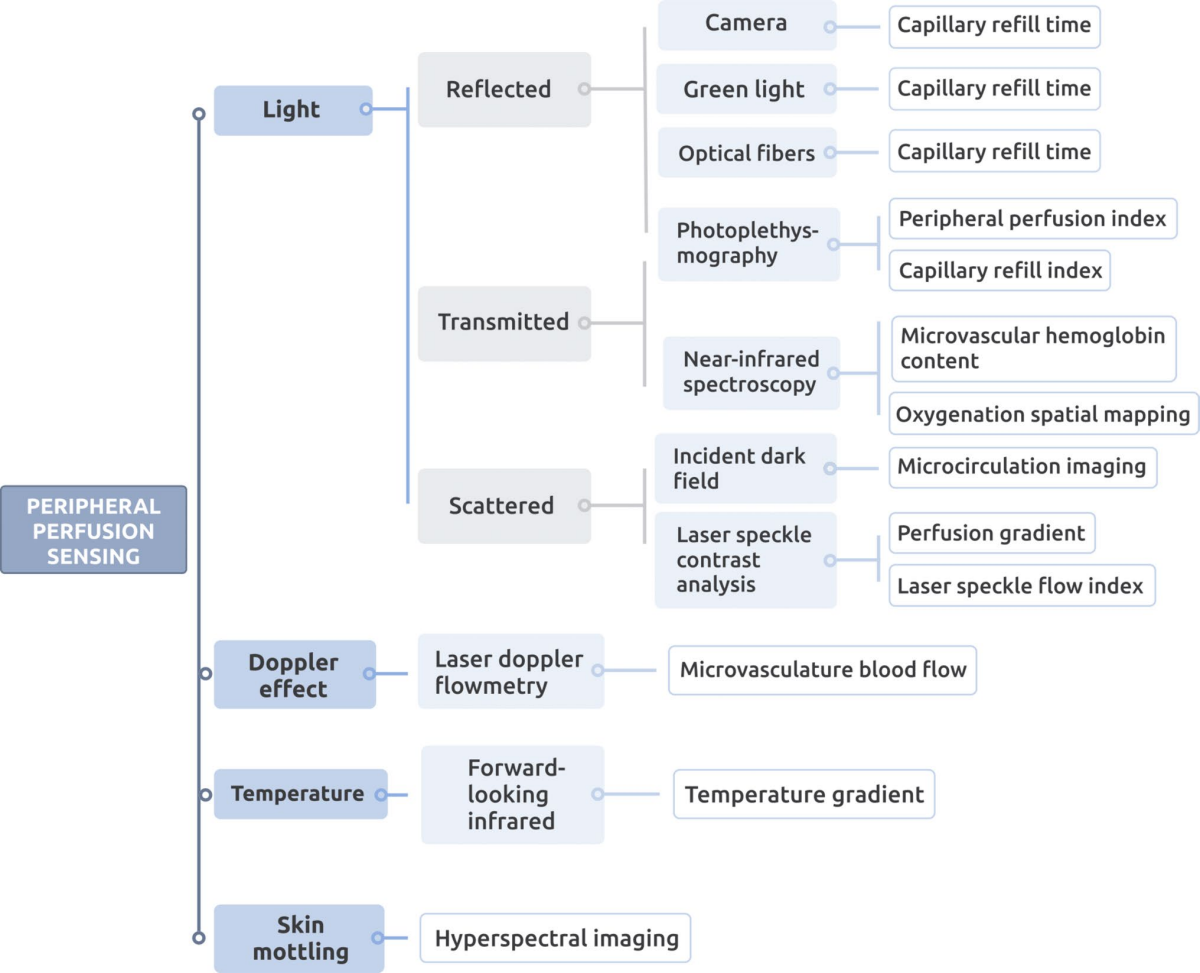
Schematic of the main non-invasive sensing technologies to monitor peripheral perfusion

The technology to non-invasively monitor peripheral perfusion can be classified based on the sensing principle in light (reflected, transmitted, or scattered), Doppler effect, temperature, and skin mottling. Figure 2 shows this classification starting with the sensing principle on the left and ending with the measured variable on the right.

### Reflected light

#### Capillary refill time

Several studies have shown the prognostic value of a prolonged CRT for the diagnosis and treatment of several conditions or diseases such as sepsis [13] or dehydration [14], and even short-term mortality [15]. The ANDROMEDA-SHOCK study proved that CRT is a better measure and guide to resuscitation than lactate levels [10,16].However, Matthias Jacquet-Lagrèze et al. [17] performed a survey of 418 adult and pediatric intensivists and found that the clinical CRT measurements were not standardized (only 3% used chronometer and 46% performed repetitions), leading to poor CRT reliability. This correlates with the moderate interobserver variability that Brabrand et al. [18] showed in their study, where nurses and nurse assistants were asked to assess the CRT as normal or abnormal. Pickard et al. [19] exposed that CRT varies with age, pressure time (lower CRT for < 3 s), pressure intensity (lower force resulted in lower CRT), pressure location (fingertip, forehead, or chest), and temperature (lower temperature increases CRT). Therefore, in order to obtain more accurate and standardized measurements, scientists are putting effort into the development of medical devices that reliably measure CRT.

Eleven medical devices were identified through the literature search; however, none are currently commercially available [20–30]. Two medical devices employ photoplethysmography (PPG) to obtain capillary refill index (CRI), a variable related to CRT [31]. CRI is the time it takes for the transmitted light intensity (TLI) to come back to 90% of its original value after releasing compression [32]. The patent research resulted in 21 patents [32–53], of which four patents correlate with medical devices found in the literature research: the hardware and pressure application method of Digital Capillary Refill device from Promedix [20], the automated quasi-continuous capillary refill timing device of Blaxter et al. [21], and the optical and pressure fiber sensor of Liu et al. [22] (included in two patents for use in the finger and foot).

The majority of these devices remain in the experimental phase. The working principle of the devices consists of applying pressure to the patient’s distal capillary bed to cause blanching. The pressure is released after some time, and the time taken by the color to return (blood comes back to capillaries) is measured. The CRT and the CRI medical devices can be classified based on the pressure application and sensing methods.

The found accuracy of some parameters used in the twelve devices are provided in Appendix A. Accuracy is not provided by all studies. Moreover, temperature, a factor that affects CRT and CRI, is not considered in some articles, decreasing the reliability of the results.

#### Pressure application method

The pressure application technique of the CRT/CRI devices can be classified as manual, pneumatic, and automatic mechanical pressure. Table 1 shows the working principle of the pressure technology following this classification.

**Table 1:** Classification of the CRT and CRI medical devices according to their pressure application method

Pressure application method			
Manual pressure	Pneumatic pressure	Automatic mechanical pressure	
		Vertical force	Swinging arm
			
[20] [22] [23] [30] [33–38]	[21] [29] [31] [40]	[26] [27] [29] [47–50]	[24] [25] [28] [41–46]

##### Manual pressure

Four papers describe medical devices for which manual pressure is applied to produce blanching [20, 22, 23, 30]. In three of the cases, the participant’s finger was pressed against the device by themselves [22] or the research assistant (RA) [20, 30]. In the study of Kviesis-Kipge et al. [23], the RA exerted pressure on a pulse oximeter finger clip where the participant’s finger was inserted. Six patents describe the use of manual pressure [35–38]. To ensure blanching, the four medical devices analyze a sensor signal. The device of Strutt et al. is a phone that vibrates and the finger’s pressure dampens the vibration. A motion sensor measures the vibration. Liu et al.’s device [22] contains a fiber Bragg grating to measure the blanching pressure and indicate when it is released. The pressure application time was 3 s in two devices in [22, 30], 4–5 s in [23], and not specified in [22]. Some patented medical devices employ a pressure sensor to calculate the applied force [33, 37, 38].

##### Pneumatic pressure

Two articles apply pneumatic pressure to the tissue [21, 31]. Both devices are composed of an air bladder that is inflated by an air pump, exerting pressure against the finger [31] or other parts of the body [21]. The device in [31], maintains the inflated bladder pressure at approximately 400 mmHg for 5 s, whereas the one in [21], applied a pressure of 130 mmHg for 7 s. Two patents describe a medical device whose pressing system consists of one [39] or two air bladders [40] that inflate and compress the tissue.

##### Automatic mechanical pressure

Six articles describe medical devices that apply automatic mechanical pressure to the capillary bed to produce blanching [24–29]. These devices push an object against the finger [25, 27], limbs [26, 29], forehead [28] or other body areas [24]. Half of the devices [24, 25, 28], apply pressure through a swinging arm. The swinging arm of Kerr et al., for example, is a finger of a robotic hand. The other devices [26, 27, 29] employ a metallic cylinder perpendicular to the capillary bed that exerts a pressure when moving downwards. The actuator of the DiCART device [26] is a piston that is moved by an electric motor. The details of the pressure application system of the other devices have not been specified. The force exerted against the tissue was 5 N in [25], and 2.8 N in [29]. Kiesser et al. [24] established the upper limit of 3.5 N for infants and 10 N for adults. The pressure was not specified in the other articles. The pressure was applied for 5 s in [25, 26, 28], 6 s in [24] and 10 s in [29].

The medical devices of ten patents apply pressure to the person’s tissue through a mechanical mechanism. Six patents make use of a swinging arm to produce blanching [41–46] In the case of CN116327159A [44], the compression is produced by an electromagnetic pressing assembly that clamps the upper and lower finger clips by suction or repulsion. The exact functioning of US2007282182A1 actuator [41] is not explained in the patent but one of the embodiments also consists of an electromagnetic pressurizing assembly. The compression system of CN217987568U [42] is composed of an active arm with a pressure sensor that moves towards a passive arm, pressing the patient’s finger: “the active arm drives the passive arm to rotate synchronously around the rotating shaft through a gear transmission mechanism”. Furthermore, medical devices of patents CN110731772A [43] and CN109171658A [46], are composed of a lever-type structure. A press plate rotates up and down around a fulcrum and compresses the tissue when going down. Finally, the compression system of CN113693570A [45] consists of a “PID pressure control system based on a steering engine and a flexible film pressure sensor”.

Four devices contain a pressing module that moves vertical toward the tissue, producing blanching [47–50] The driving force of the module movement in the patent CN210446999U [47] is an inflatable airbag, whereas it is not explained in the patents [48, 49]. The patent KR101459652B1 [50] consists of two plates and a rotatable component. By rotating the outer component, the two plates are drawn together, resulting in compression.

The pressure application method in the other four patents is not explained. Patent US2013018241A1 [51] exposes that their invention comprises “means to momentarily exclude blood from or reduce blood flow to the measurement region” and that means may be a convex surface. Patents US2023048928A1 [52] and JP2012115640A [53] just mention that the tissue is compressed but not how. Finally, it is written in patent GB2596913A [54] that the pressure may be applied pneumatically or hydraulically.

#### Sensing technology of CRT devices

The technologies used to sense CRT can be divided into digital camera, polymer optical fiber, and PPG. Table 2 shows this classification.

**Table 2:** CRT devices classification according to their sensing technique

Sensing technology	Articles	Patents
Digital camera	[24] [25] [26] [28] [29]	JP2020110616A [34] US11712165B2 [35]
Polymer optical fiber	[22]	US2021251571A1 [37] WO2015155526A1 [38]
PPG	[20] [21] [23] [27] [30]	WO2017009669A1 [39] CN217987568U [42] CN113693570A [45]

##### Digital camera

Five devices employ a digital camera placed above the tissue without touching it, to calculate CRT [24–26, 28, 29]. The camera starts recording when the pressure is released [24–26], or a bit before [28, 29] until 6–20 s after pressure release [26, 28, 29]. In order to avoid the effect of environmental light variations Kieser et al. [24], Ruste et al. [26], and Bachour et al. [29]. employed LEDs to illuminate the scene. Bachour et al.l [29]. included cocircular polarizers between the camera and LEDs to attenuate the light reflected on the outer surface of the skin.

##### Optical fibers

One device uses polymer optical fibers (POF) to analyze reflected light and calculate CRT [22].

##### PPG-reflected light

PPG can measure changes in blood volume by detecting variations in light reflection. Five devices employ PPG to sense light absorption variations and calculate CRT [20, 21, 23, 27, 30]. The main difference between them is the type of light that they use to illuminate the capillary bed and the detector. Blaxter et al. [21], developed a sensor composed of a ring of 6 LEDs with wavelengths of 950 nm, 640 nm, and 520 nm, and a photodiode. Kviesis et al. [23] employed a system consisting of a blue LED and a blue-photosensitive photodiode to measure CRT. Two devices illuminate the capillary bed at 525 nm (green light) and record the optical waveform produced from the light reflectance [20, 27]. CRT is calculated using signal-processing algorithms. The Digital Capillary Refill device of Promedix [20] contains a mobile application that calculates the CRT in real-time. The mobile application CapApp [30], illuminates the capillary bed with the phone screen light and detects the color change with the internal phone camera when the finger is pressed against it.

Some difficulties have been encountered in determining the optical method employed for the patented devices to detect CRT. The information presented by most of the patents is vague. For example, patent US11806114B2 [33] claims a medical device comprising an “optical sensor configured to generate an optical signal representative of optical energy received from an optical source over time”, without providing more details about the type of optical sensor used. Moreover, several patents [36, 40, 47, 54,49] just mention that they use a sensor that detects tissue color change or blood refilling time. Other patents [41, 44, 50, 51], although they do not fully explain the sensing technique, include the use of a light source that emits radiations with two different wavelengths.

#### Near-infrared spectroscopy

Near infrared (NIR) spectroscopy can *also* be used in imaging techniques to generate a spatial mapping of tissue perfusion. SnapshotNIR is a hand-held reflectance multispectral imaging system that uses various LED flashes in the red and near-infrared electromagnetic region to generate a tissue oxygenation map based on the relative concentration of oxy- and deoxyhemoglobin [55]. NIROS [56] generates near-real-time spatio-temporal tissue oxygenation maps. This device uses 4 multi-wavelength LEDs (690 nm0, 830 nm) to obtain hemoglobin-based parameters from the diffuse reflectance signals and produce dynamic images (1 Hz per wavelength) of the tissue perfusion.

### Transmitted light

#### PPG-Transmitted light

Photoplethysmography can also detect changes in peripheral circulation by detecting variations in light transmission. Shinozaki et al. [31] obtained the CRI using the infrared wavelength (940 nm) of a pulse oximeter.

The variable peripheral perfusion index (PFI) derived from pulse oximetry can be used as an indicator of peripheral perfusion. PFI is the ratio between the pulsatile and non-pulsatile components of the pulse oximeter [56]. This ratio is principally affected by vascular tone and blood flow. Therefore, PFI is a sign of the state of the sympathetic/parasympathetic nervous system balance and cardiac output. Studies have shown the relationship between PFI and other peripheral perfusion predictors. A decrease in PFI, for example, is correlated with an increase in core-to-toe temperature difference [58] and an increase in CRT [59]. Although there is no standard PFI value to distinguish normal from abnormal perfusion, Lima et al. [58], established 1.4 as the limit, and Iizuka et al. [59] proposed that a ratio smaller than 1.8 corresponds to poor peripheral perfusion. Since a pulse oximeter is already available in ORs and ICU, this variable can be employed to continuously monitor peripheral perfusion, also during anesthesia when CRT is more difficult to measure [59]. However, the PFI values in the population are skewed and therefore it is more reliable to evaluate changes within the same person [57].

#### Near-infrared spectroscopy

NIR employs the near-infrared region of the electromagnetic spectrum (780–2500 nm) to determine tissue oxygen uptake by monitoring the difference between deoxyhemoglobin and oxyhemoglobin [60]. Generally, a NIR probe is composed of a light source and two photodetectors at different locations to measure tissue oxygen uptake at different depths (not absorbed light returns to the photodetector) [60, 61]. The difference between the returned light from the deep minus the surface path represents the tissue bed spectral absorption.

Mendelson et al. [62] proposed the measurement of microvascular microhemoglobin content (MHC) using high-resolution NIR spectroscopy as an alternative to the total hemoglobin content. They measured the temporal change in the optical density of MHC at 798 nm (isosbestic hemoglobin wavelength), showing the dynamic hemoglobin distribution variability in the microcirculation. Lin et al. developed an assistance system for arteriosclerosis [63] and diabetes [64] evaluation based on peripheral perfusion monitoring. They made use of NIR spectroscopy (wavelengths 640, 700, and 910 nm) combined with a sphygmomanometer to monitor the change in hemoglobin parameters for different pressures in the arm.

### Scattered light

*Laser speckle contrast analysis* (LASCA) is an optical technique that consists of the illumination of the tissue by a laser [65]. The backscattered light produces an interference pattern (speckle pattern). Different degrees of movement from the tissue particles (red blood cells) generate different degrees of blurring in the speckle pattern (more blurred with more movement). This spatial blurring is quantified by calculating the image contrast, giving rise to a contrast image whose perfusion values are inversely proportional to the contrast. Therefore, by using LASCA over the skin, a spatio-temporal peripheral perfusion map can be obtained. Ruaro et al. [66] used that map to successfully evaluate peripheral blood perfusion in systemic sclerosis patients obtaining results that correlate with nailfold videocapillaroscopy results. Bonetta-Misteli et al. [67] employed laser speckle flow index (LSFI), a LASCA-derived variable to predict postpartum hemorrhage. Hemorrhage is characterized by some compensatory processes such as peripheral vessels’ vasoconstriction. Therefore, by continuously monitoring LSFI, which is directly proportional to blood flow, hemorrhage can be detected.

### Doppler effect

*Laser Doppler flowmetry* (LDF) can measure microcirculatory variables and detect fast perfusion changes, by measuring the Doppler shift produced by red blood cells when they are illuminated [68]. The skin lights up by a laser and the fluctuations generated by the reflected light provide information about red blood cell concentration, velocity, or flux. Mongkolpun et al. [69] showed that LDF has the potential to monitor tissue perfusion in circulatory shock by measuring skin blood flow during thermal challenge (capillary vasodilation due to temperature increase) in healthy volunteers and patients with circulatory disease. The degree of skin blood flow alterations detected by LDF was proportional to the lactate levels and Sequential Organ Failure Assessment (SOFA) score. Cutolo et al. [70], assessed fingertip blood perfusion in systematic sclerosis patients using LDF and found a negative correlation with the microvasculature damage visualized with nail-fold video capillaroscopy, indicating that it can be used for an early diagnosis. Note however, that this technology is influenced by the tissue’s optical properties and motion noise. Moreover, the results are relative measurements.

### Temperature

*Forward-Looking Infrared*. Infrared thermography measures the infrared radiation emitted from any non-contact object [71]. Forward-Looking Infrared (FLI) thermal imaging camera can be used to visualize temperature gradients [24, 71–73]. Peripheral vasoconstriction during shock results in a decrease in skin temperature due to reduced blood supply in peripheral tissues, while the core body temperature remains constant. Therefore, central-peripheral temperature difference can be used to assess peripheral perfusion [57].

Kazune et al. [71] measured skin temperature in the knee and upper thigh in septic shock patients and they found that lower skin temperature was associated with a high mottling score, another peripheral perfusion evaluation sign. Kieser et al. [24] evaluated peripheral perfusion using CRT and skin temperature sensors and obtained similar results for the hydration degree (CRT and skin temperature increases for higher). Moreover, Wallace et al. [72] employed smartphone-based FLIR cameras (FLIR One Personal Vision System for iOS) to obtain thermographs of each extremity to calculate ankle-brachial index (ABI). ABI is a method to evaluate extremity perfusion by dividing the higher extremity pedal pressure by the higher extremity brachial pressure. The brachial and dorsalis pedis pressures were obtained using a handheld doppler probe. They found that the thermal ABI (highest temperature of lower extremities divided by the highest temperature of upper extremities) correlates with the traditional ABI and therefore, smartphone-based FLIR cameras can be used to assess extremities perfusion. Luo et al. [73], developed a deep learning algorithm to predict mortality risk in critically ill patients based on the analysis of infrared thermography images. They found correlations with conventional perfusion parameters and the hypoperfusion severity. Note that measurements can be affected by variations in ambient temperature or hypothermia[74].

### Skin mottling

Skin mottling is a temporal skin condition characterized by a mottle, red, blue or violaceous reticulate pattern in the skin as a result of heterogeneous small vessel vasoconstriction [75, 76]. In clinical practice, the mottling severity is visually assessed around the knee with a six-grade scoring system where 0 corresponds to “no mottling”, and 5 to “extensive mottling reaching above the groin fold”. To quantify mottling with a more accurate method, Saknite et al. [75] employed hyperspectral imaging (HSI). They developed an algorithm that calculates the skin chromophore concentration and oxygen saturation based on the assumption that the skin is composed of two layers (epidermis with melanin and dermis with blood). The light is scattered in both layers but absorbed by oxy-, deoxyhemoglobin, and melanin in the epidermis. By applying the inverse diffusion model, the average saturation was calculated for each part of the tissue. Using HSI to measure oxygen saturation allows earlier diagnosis of microcirculatory alterations than visual mottling assessment. One limitation of mottling as a sign of peripheral perfusion alteration is that its detection is limited in people with dark skin.

### Technology combination

In order to obtain more precise and reliable knowledge about the perfusion status and be able to better predict different disorders and diseases, some experts have opted for the combination of these technologies to obtain better specificity and sensitivity. For example, Ovadia-Blechman et al. [77], combined Doppler flowmetry, PPG, and transcutaneous oxygen tension monitor to measure peripheral microcirculatory hemodynamics during different degrees of hypoxia. This system monitors peripheral tissue oxygenation, as well as changes in central oxygen pressure during hypoxia with high sensitivity. Shaknite et al. [75] showed that HIS combined with thermal imaging provides clinically valuable information related to peripheral tissue oxygen saturation and its heterogeneous distribution in septic patients. Kieser et al. [24], who developed and compared CRT, skin temperature and skin recoil sensors, concluded that combining the 3 different markers using continuous values for the predictors and linear regression achieved better dehydration prediction performance than any of the individual indicators.

## Discussion

The state of the art of different technologies for monitoring peripheral perfusion has been analyzed. They measure different targets and generate different types of results (e.g., variation of a variable over time, ratios, spatiotemporal maps). The main limitation of these technologies is that there are no standard values for adequate or inadequate peripheral perfusion, as little data about the variables variation within population and conditions are available. Hence, more research has to be performed to correlate the alterations in peripheral perfusion with other physiological variables and different diseases and conditions.

For the measurement of peripheral perfusion using transmitted light, often already commercially available devices, such as pulse oximetry or NIR spectroscopy, have been used, making it possible to use them more easily on patients in a clinical setting. Although many of the technologies have shown their potential to detect microcirculation alterations, still further research has to be conducted in patients, e.g., to evaluate whether using these technologies has a meaningful effect on morbidity and/or mortality. Furthermore, ideally, this technology should be able to be used worldwide in all hospitals. Therefore, it should be compatible with other hospital equipment and setups, and its cost should not be too high, in order to be affordable also in low and middle income countries.

A substantial amount of the technologies are still in the experimental phase, and many of the medical devices have been tested only in healthy subjects. This is particularly true for devices that use transmitted light and controlled (non-manual) compression techniques for CRT measurements. The authors are not aware of a commercially available device to measure CRT objectively with controlled compression, but it is possible that some companies are working (under the radar) on commercializing such a CRT device. Moreover, from patents and literature, it was difficult to assess the Technology Readiness Levels (TRLs) of many of these experimental devices. In general, patents do not provide information on the clinical utility or accuracy of the proposed methods, and only a limited number of published articles (Table 3) report e.g. accuracy of CRT data, and these are also restricted to studies in healthy individuals.

The development from prototype to a commercial product is often however a long and costly process. The Medical Devices Regulation (MDR) and Food and Drug Administration (FDA) e.g., ask for many investigation and administrative steps, before a device can be classified as certificated medical device. This include e.g. a risk analysis, a quality management system, technical documentation, a certificated manufacturer, and finally a clinical evaluation and post-market surveillance. This is a long and expensive trajectory. Therefore, in practice many patented devices will never reach the market. The increasing number of articles and patents on devices to measure peripheral perfusion, however, clearly indicate the need for a clinically useful objective peripheral perfusion measurement device.

## Discussion on the technologies

CRT is already employed in clinical settings for the assessment of circulatory status, sepsis evaluation, trauma assessment. Therefore, the incorporation of a device that evaluates peripheral perfusion by measuring CRT would not entail extra training for health professionals. Moreover, a device that measures CRT would avoid the subjectivity and interobserver variability that are present in manual CRT, producing more reliable results. Furthermore, if a CRT device was widely used in different hospitals with a large number of patients, CRT values could be standardized depending on the patient’s characteristics, such as age and sex. This would produce even more reliable results that could have a great impact on the early diagnosis of diseases. Also, CRT should be further investigated to establish standard values and determine how factors such as temperature, pressure force, and duration affect it. Some of the medical devices employ a modified pulse oximeter that is already present in hospital rooms. Other CRT devices are bulky and not compatible with current hospital technology.

The devices with manual pressure are cheaper and simpler than the other devices. However, this method also generates more variability between measurements and less reliable results. Moreover, in order to generate an optimal diagnosis CRT results should be calculated in real time. However, most of the devices collect the CRT/CRI data and process them later. In the future, AI methods could be used for signal analysis and decision making, but for this a lot of data is required. At this moment, the majority of the experiments were performed to prove the device concept so the sample size was small and not much data is available regarding accuracy. Furthermore, CRT varies with age and therefore, results of CRT measurements of different medical devices performed in different age groups are not fully comparable [19]. Also, infant participants tend to move, generating motion artifacts that affect the results [24, 30]. Moreover, the sample size of some experiments is low, which can result in reduced statistical power, limited generalizability, and increased variability and risk of bias.

NIRS has the advantage of allowing continuous and real-time monitoring of the peripheral perfusion condition [60]. It can also provide tissue oxygenation measurements and therefore, by using a single device, information about different parameters could be obtained. Moreover, in determined situations, a spatiotemporal map can provide information more relevant than the variation of a variable over time. Nevertheless, visualization is less precise than quantitative measurements. Furthermore, this technology is not readily available in every ICU and can be expensive to purchase and maintain.

PFI provides real-time information about microcirculation alterations and detects changes in peripheral perfusion quickly. Because it is a value derived from pulse oximetry, it can be measured at the bedside using a standard pulse oximeter. Moreover, it is easily interpretable, as it is just a number relating the strength of the pulsatile component compared to the non-pulsatile. However, PFI is affected by the autonomic activity and the cardiac output [57]. Therefore, PFI can only be measured in conditions in which only one variable varies. Moreover, this technology’s validation is limited in certain patient populations and clinical scenarios.

Laser Doppler flowmetry can dynamically monitor microcirculatory alterations and therefore, predict circulatory diseases. However, LDF does not provide absolute perfusion values but relative ones [70]. Furthermore, this technology has been mainly used in systematic sclerosis and it is under research for other pathologies.

Temperature gradients provide information about microcirculatory alterations during shocks. Nevertheless, they use extra equipment that can be bulky and not compatible with the hospital setup. Moreover, thermographic images can be difficult to interpret and generate relative results. Also, temperature gradients are affected by different factors such as hypothermia or ambient temperature.

There is almost no technology that quantitatively measures skin mottling (only one article was found). This could be due to the subjectivity of this parameter when assessed visually, its potential for misinterpretation (e.g., it could be confused with pressure injuries, skin pigmentation, or bruising), and its limited detection in people with dark skin.

Although the exposed technologies have shown their potential for monitoring peripheral perfusion, it has been proven that the combination of several technologies has an even better prognosis value for shock diagnosis and treatment. The further combination of several of those technologies for peripheral perfusion with macrocirculatory parameters (venous saturation, blood pressure, cardiac output…) could be the key to an early shock or other diseases’ diagnosis and treatment.

## Conclusions

Microcirculatory alterations are a strong predictive factor of shock development and outcome. Different technologies have been found to monitor peripheral perfusion, but they still need more research and testing under different conditions before they can be used in clinical practice. The combination of technology that monitors microcirculatory with macrocirculatory parameters has the potential to have an accurate prognosis value for shock and other diseases.

**Table 3:** Summary of the accuracy of the twelve medical devices measuring CRT

Ref.	Method	*N* particip.	Age (years)	*N* rep.	Pressure	Temperature	Accuracy
[20]	CRT measured with the device and compared with manual CRT and lactate levels	563 (different septic levels)	49.1 (mean)	3	Manual (device app for magnitude and duration)	Not considered	Specificity on sepsis outcome: 73% Sensitivity: 40–100%
[21]	Participants’ arm were cooled from ambient temperature to 5 °C and CRT was measured with the device	30 (healthy)	15 adults 15 children (age 5–15)	CRT every 23 s (922 refills)	130 mmHg	Thermistor	No definitive conclusions due to limited amount of data. Statistically significant change of CRT with temperature
[22]	Participants press their index finger against the sensor	10 (healthy)	-	10	Manual (until no PPG signal) and FBG pressure sensor	Constant room temperature (24–26 °C)	-
[23]	Participant’s finger was compressed, and CRT was measured	11 (healthy)	18–31	10 per finger (forefinger and little finger)	Manual (until no PPG signal)	Not considered	-
[24]	CRT measured with the device in participants with different dehydration severities	19 (healthy)	9 adults (mean 23.4) 10 infants (mean 10 months)	-	Constant (max 10 N for adults, 3.5 N for infants)	Not considered	Specificity on hydration level: 50% Sensitivity: 50%
[25]	CRT measured with the device different days to investigate effects of the imaging mode and start time on CRT precision	6 (healthy)	22–23	4	5 N	Constant room temperature (25 °C)	-
[26]	CRT measured with the device before and during venous and arterial occlusion tests and compared with manual CRT	15 (healthy)	24–27	3 in upper and lower limbs	Constant by the device	Constant room temperature	Specificity: 92% Sensibility: 91%
[27]	CRT measured with the device using passive leg raising test and fluid challenge, and compared with manual CRT	8 (ARDS due COVID-19)	-	-	Constant by the device	Constant room temperature (22 °C) and temperature sensor	The detected CRT showed decreased time during fluid resuscitation, similar to the manual CRT method
[28]	CRT measured with the device in the forehead and compared with manual CRT	12 (healthy)	18–65	3	Constant by the device	Not considered	Detected CRT corresponds to the same category (normal or prolonged) as manual CRT)
[29]	CRT measured with the device in the forearm in different skin phenotypes	22 (healthy)	20–70	5	2.5 N	Constant room temperature (20–22 °C)	The values for measured CRT are larger than for manual CRT
[30]	CRI measured with the device in right and left index fingers and compared with manual CRT	152 (healthy)	0–9 (*n* = 42) 10–12 (*n* = 48) 13–15 (*n* = 34) 16–17 (*n* = 28)	Until satisfactory signal or 3 times	RA presses participant’s finger against device	Not considered	CRI results positively correlate with standard clinical CRT, but manual CRT demonstrated improved consistency over CRI
[31]	CRI measured with the device and compared with manual CRT	30 (healthy)	23–61	10	400 mmHg	Constant room temperature (20–22 °C) and thermocouple	High intra-rater reliability (0.97). Bland-Altman plots suggest systematic bias (CRI consistency higher than CRT + 1.01s). Strong correlation between CRI and CRT (*r* = 0.89, *p* < 0.001)

## Data Availability

No datasets were generated or analysed during the current study.

## Appendix A

See (Table 3)
